# Single-cell RNA sequencing reveals changes in glioma-associated macrophage polarization and cellular states of malignant gliomas with high AQP4 expression

**DOI:** 10.1038/s41417-022-00582-y

**Published:** 2023-01-04

**Authors:** Ran Wang, Lu Peng, Yong Xiao, Qi Zhou, Zhen Wang, Lei Tang, Hong Xiao, Kun Yang, Hongyi Liu, Li Li

**Affiliations:** 1 Department of Neurosurgery, The Affiliated Brain Hospital of Nanjing Medical University, Nanjing, Jiangsu China; 2 Department of Clinical Laboratory, The Affiliated Brain Hospital of Nanjing Medical University, Nanjing, Jiangsu China; 3 Department of Neuro-Psychiatric Institute, The Affiliated Brain Hospital of Nanjing Medical University, Nanjing, Jiangsu China; 4 Department of Laboratory Medicine, Shanghai General Hospital of Nanjing Medical University, Shanghai, China

**Keywords:** CNS cancer, Tumour heterogeneity

## Abstract

Glioma is the most common primary central nervous system tumor in adults. Aquaporin-4, as a water channel protein encoded by AQP4 in the brain, is reported to alter its aggregation status to affect plasma membrane dynamics and provide the potential for metastasis of tumor cells and components of the tumor microenvironment. We performed single-cell RNA transcriptome sequencing of 53059 cells from 13 malignant glioma samples and spotted that the expression of AQP4 differed between samples. The same result was observed in the TCGA glioma database, showing poor overall survival and poor response to chemotherapy in AQP4 overexpressed populations. Concomitant with the overexpression of AQP4, genes related to the immune system were also over-expressed, such as CD74, HES1, CALD1, and HEBP2, indicating AQP4 may relate to immune factors of tumor progression. We also found that tumor-associated macrophages tended to polarize toward M2 macrophages in the high AQP4 group. In glioblastoma samples, we examined cell status differences and identified that cell status differs according to AQP4 expression levels. Briefly, our study revealed substantial heterogeneity within malignant gliomas with different AQP4 expression levels, indicating the intricate connection between tumor cells and the tumor immune environment.

## Introduction

Glioma is the most common type of tumor in adults’ central nervous system (CNS). Grade III and IV gliomas are more malignant due to their rapid progression and high recurrence rate [1]. Glioblastomas (GBMs), in particular, lead to a poor prognosis despite the standard radiotherapy and chemotherapy administered, with overall survival of only 12 to 18 months [2].

Targeted therapies have always been potential strategies for this notorious tumor. However, it has had little impact on glioma treatment these years. Recent advances [3, 4] suggest that targeted therapies that are often used in clinical practice won’t benefit overall survival as they may induce resistance after a period of therapy, leading to immune suppression. Immunotherapy strategies are also meant to deal with this tricky situation. However, evidence shows that the efficacies of immunotherapy remain limited, including cellular [5, 6], vaccination [7, 8], and immune-checkpoint inhibitor treatment [9].

The tumor microenvironment (TME) consists of immune cells, lymphocytes, bone marrow-derived inflammatory cells, blood vessels, extracellular matrix, fibroblasts, and signaling molecules [10]. TME, especially the immune microenvironment, is associated with tumorigenesis as it can harbor tumor cells and stimulate uncontrolled cell proliferation [11–13], explaining the poor efficacy of current treatments.

Recent studies show that the immune microenvironment varies across different tumor types. Unlike nuances of cell atlas in melanoma, pancreatic, colorectal, and breast cancer, GBMs seem to change the composition of the immune microenvironment significantly to the opposite extreme [14], indicating potential characteristics related to the immune microenvironment are to be discovered.

The lymphatic system is the bridge between the circulatory and the immune system. It has been recently identified that the brain has its unique lymphatic system—the glymphatic system. It clears the interstitial solutes in the brain parenchyma, associated with the causes of many neurodegenerative diseases [15, 16]. The glymphatic system clears the interstitial solutes in the brain parenchyma. Moreover, it has been revealed to transport CNS-derived antigens to induce an immune response, influencing the outcome of brain tumors [17]. Aquaporin-4 (AQP4) is a crucial protein in the glymphatic system [18]. It is concentrated in perivascular and subpial end-foot membranes, and creates micro-domains at the blood-brain and cerebrospinal fluid brain barrier [19]. The previous study [20] shows that the expression of AQP4 varies across different grades and individuals. Due to its pivotal role in the glymphatic system, AQP4 may contribute to the impact on glioma malignancy. At present, some studies suggest AQP4 may be a marker for the progression of malignant glioma [21], while others argue that AQP4 has no impact on the overall survival of IDH-wildtype GBMs [22]. As for immune-related aspects, there is little research regarding AQP4 and glioma’s immune system within the last ten years except for two case reports [23, 24]. Its role in the glioma immune microenvironment is yet to be revealed.

Single-cell RNA sequencing (scRNA-seq) allows us to probe the expression distribution down to the individual cell level [25]. As the diversity of cell type and tumor microenvironment is increasingly studied in the field of glioma therapy, scRNA-seq becomes more and more appealing to glioma research. By digging and comparing the heterogeneity of glioma cells based on the expression of AQP4, we investigated the correlation between AQP4 and the glioma immune microenvironment from a novel perspective.

## Results

### Tumor heterogeneity revealed within glioma samples

**Fig. 1:**
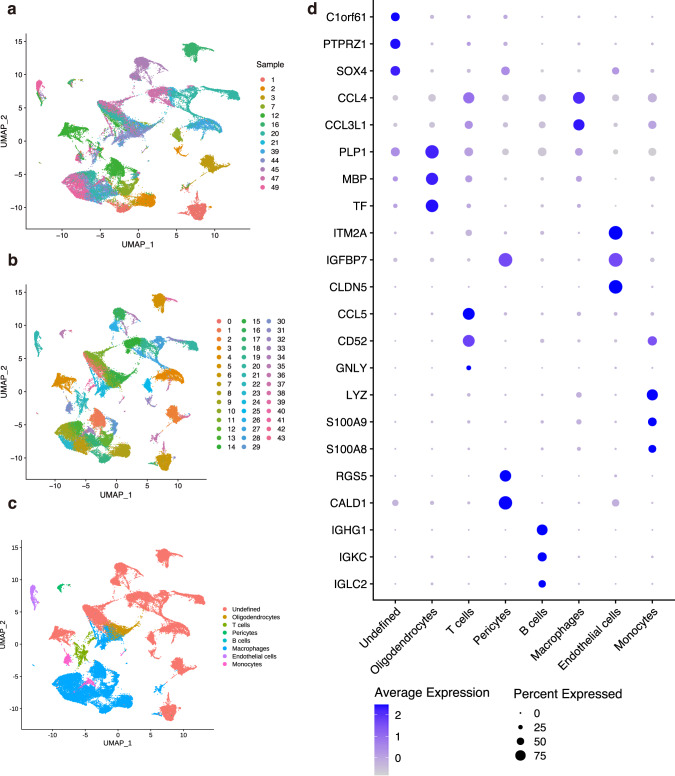
scRNA-seq result of 13 individual glioma samples. **a** The UMAP plot showed 53059 profiled cells from glioma samples. Different samples were labeled with different colors. **b** Unsupervised clustering results distinguished with different colors. **c** Cell types are marked with different colors. **d** Dot plot demonstrated three top expressed genes of each cluster. The color of dots represents the mean level of a specific gene, and the size of dots represents the number of cells expressing this gene.

We used the public glioma dataset GSE135045 [26], and collected additional resection samples from 6 patients with malignant glioma during surgery. All glioma samples used were assessed by pathologists using the 2021 WHO Classification of Tumors of the Central Nervous System (Supplementary Table S1). After constructing scRNA-seq libraries according to the protocol, we performed strict quality control, normalization, and data scaling. Eventually, we obtained a total of 53059 cells and 34031 genes per cell for subsequent analysis (Fig. 1a). These cells were clustered into 43 groups using unsupervised Uniform Manifold Approximation and Projection (UMAP) (Fig. 1b). Two comprehensive reference data sets were used to perform cluster annotation (see Material and methods). We managed to confirm the identity of macrophages, monocytes, T cells, B cells, oligodendrocytes, and pericytes (Fig. 1c, d). The rest of the cells were suspiciously malignant cells as these clusters were far away from each other, suggesting that they were highly heterogeneous as formerly described [27].

### AQP4 expression profile varies across glioma samples

**Fig. 2:**
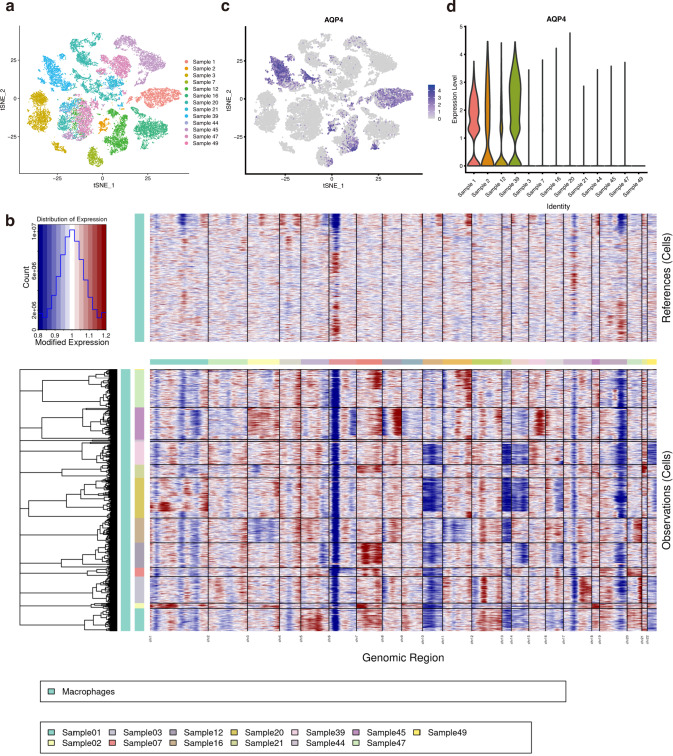
AQP4 expression profile in malignant glioma cells. **a** The potential malignant glioma cells were divided based on t-SNE analysis. **b** The heatmap demonstrated the CNV profiles inferred scRNA-seq data for all likely malignant glioma cells. The colored bar describes different chromosomes on top and samples on the left. All undefined cells exhibit apparent chromosome gain (red) or loss (blue), compared to macrophages. **c** The feature plot highlights the AQP4 expression profile in malignant glioma cells. **d** Violin plots displaying AQP4 across clusters of samples.

Assuming those undefined clusters of cells are genuine tumor cells, we extracted these cells and performed unsupervised clustering using t-Distributed Stochastic Neighbor Embedding (t-SNE) for visualization (Fig. 2a). As a result, we found it still remarkable that the samples were highly different. Therefore the influence of other types of cells on clustering was excluded. To further verify whether these cells are genuine malignant cells, we analyzed the copy number variation (CNV) of each sample. We use the inferCNV R package to infer somatic large-scale chromosomal copy number alterations [28]. According to inferCNV manual, the cut-off was set to 0.1 for the minimal average read counts per gene among cells. The output heatmap (Fig. 2b) was rather apparent that all samples showed over or less abundance in different regions of the tumor genome, respectively. Thus, we were confident that these previously undefined clusters of cells were tumor cells.

Based on the dimensional reduction plot of tumor cells, we further examined the AQP4 expression among each glioma sample. Interestingly, the AQP4 expression profile varies across glioma samples. The expression of AQP4 was relatively high in 4 samples (Sample 1, 2, 12, and 39) and low in the other samples (Fig. 2c, d). For the convenience of subsequent analysis, we put samples 1, 2, 12, and 39 together as the “high AQP4” group, samples 3, 7, 16, 20, 21, 44, 45, 47, and 49 as “low AQP4” group.

### The association between AQP4 expression and the prognosis of gliomas

**Fig. 3:**
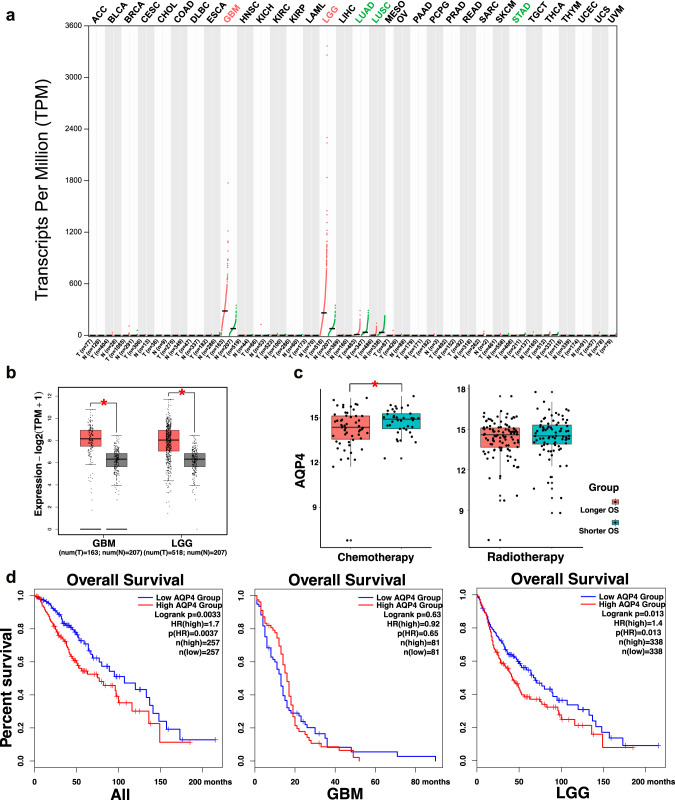
Landscape of AQP4 expression in TCGA dataset. **a** AQP4 expression profile across all tumor samples and paired normal samples. Tumor samples (T) are indicated by red, and normal tissue samples are green, using a normalization method that calculates transcripts per million (TPM). **b** The TCGA LGG (*n* = 518) and GBM (*n* = 163) dataset, as well as GTEx normal brain tissues data (*n* = 207) showing AQP4, was upregulated in tumor samples. **c** The AQP4 Kaplan–Meier survival analysis shows OS of all gliomas (left), GBMs (middle), and LGGs (right). **d** Boxplot displaying AQP4 expression levels of longer and shorter OS groups in samples went through chemotherapy (left) and radiotherapy (right).

To demonstrate the meaning of AQP4 expression diversity, we used the TCGA GBM (the glioblastoma dataset, *n* = 163), LGG (the lower grade glioma dataset, *n* = 518), and GTEx normal brain tissues data (*n* = 207) to compare with all types of tumor samples and paired normal tissues. The gene expression profile showed that AQP4 is highly expressed in gliomas (including GBM and LGG) than in normal brain tissues. In other organs, comparably, AQP4 expressions are at very low levels in both tumor and paired normal tissues (Fig. 3a), consistent with previous studies [29, 30]. We further compared AQP4 expression levels between tumor and normal brain tissues of LGG and GBM, respectively, and found that AQP4 tends to be overexpressed in the tumor samples (*p* < 0.05, Fig. 3b), inferring that AQP4 may have an impact on glioma development. The Kaplan–Meier survival analysis was performed to investigate the association between AQP4 expression and prognosis of gliomas to describe the Overall Survival (OS) curves of AQP4 over- and lower-expression groups in all gliomas (TCGA GBM and LGG), GBM, and LGG datasets, respectively. Log-rank was used to validate the difference between two groups in each dataset (Fig. 3d). The hazard ratio was calculated based on the Cox proportional-hazards model to evaluate the risk of death. Compared to the low AQP4 group, the high AQP4 group tended to have shorter OS in all gliomas (Fig. 3d, Supplementary Fig. S1). In LGG, the same result was achieved. In GBM, however, the discrepancy of OS was not significant between low AQP4 and high AQP4 groups, consistent with the result of an earlier study [22].

Previous studies have shown that AQP4 regulates water homeostasis in the brain [31, 32], potentially associated with blood-brain barrier permeability and cerebral edema. Cerebral edema comes with radiotherapy [33, 34], leading to a poor prognosis. On the other hand, Blood-brain barrier permeability can affect chemotherapy efficiency [35–37], leading to a poor prognosis. Thus, we continued to investigate its correlation with radiotherapy and chemotherapy prognosis. We acquired data from glioma individuals who went through radiotherapy (*n* = 184) and chemotherapy (*n* = 93) from the TCGA dataset (Supplementary Table S2) for subsequent analysis. Median OS was used as a cut-off where individuals with larger values were assigned to the Longer OS group and the others to the Shorter OS group. We compared AQP4 expression levels between two groups and found that individuals with shorter OS in the chemotherapy group showed higher AQP4 expression levels than those with higher OS (Fig. 3c, Supplementary Table S3). On the other hand, this difference was not significant in the radiotherapy group, suggesting that AQP4 was more meaningful for cases that went through chemotherapy rather than radiotherapy.

### AQP4 related differentially expressed genes are associated with glioma immune microenvironment

**Fig. 4:**
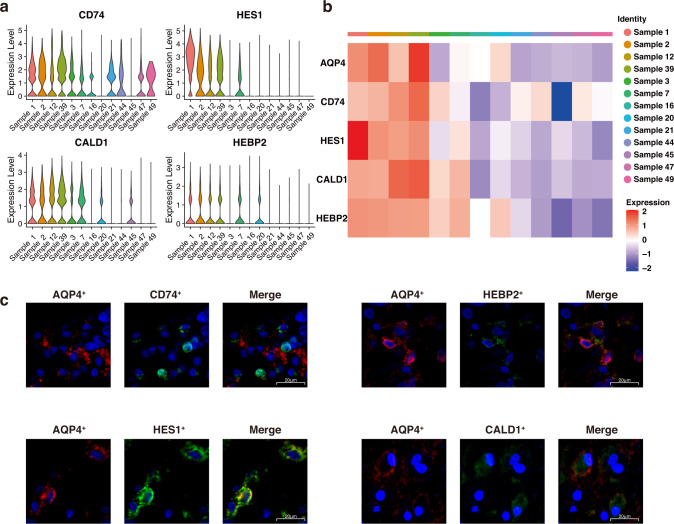
Differential expressed genes related to AQP4 expression. **a** Violin plots displaying AQP4-related DEGs expression levels across single-cell samples. **b** Average expression level of AQP4-related DEGs of all single-cell samples. **c** Representative immunofluorescence staining images showing the expression of AQP4 and its DEGs in patient tumor sample. Scale bar: 20 μm.

We next focused on AQP4-related genes, trying to discover events that would potentially influence the prognosis of glioma. “FindAllMarkers” function of the Seurat R package was used to perform the Wilcoxon Rank Sum test to identify differentially expressed genes (DEGs) that were upregulated in each group (Supplementary Table S4). Gene ontology analysis showed that genes upregulated in the high AQP4 group are relevant to immune-related entries (Supplementary Fig. S2). Eventually, we targeted four over-expressed genes (Fig. 4a, logged fold change >0.25) in the high AQP4 group: CD74, HES1, CALD1, and HEBP2. The average expression of each DEG was also calculated in non-log space by Seurat’s “AverageExpression” function, showing the notable difference between the two groups (Fig. 4b, c). A recent study showed that brain tumors utilize CD74 activation to escape pro-inflammatory M1 conversion [38], indicating that CD74 is closely related to the glioma immune microenvironment. HES1 has been proven regulated by the NOTCH signaling pathway [39], along with the Hedgehog and Wnt signaling pathways [40], which are common pathways of tumor cells and also related to immune response [41]. CALD1 is a cytoskeleton-associated protein that has been revealed to be related to neoplastic angiogenesis [42], and immune infiltrates in gastric cancers [43]. As for HEBP2, there is not enough evidence showing its correlation with tumor cells, but it is believed that its related pathways are innate immune system [44]. All four selected DEGs were either related to the microenvironment or the immune system, which is thought-provoking that these DEGs and AQP4 may be associated with the glioma immune microenvironment.

**Fig. 5:**
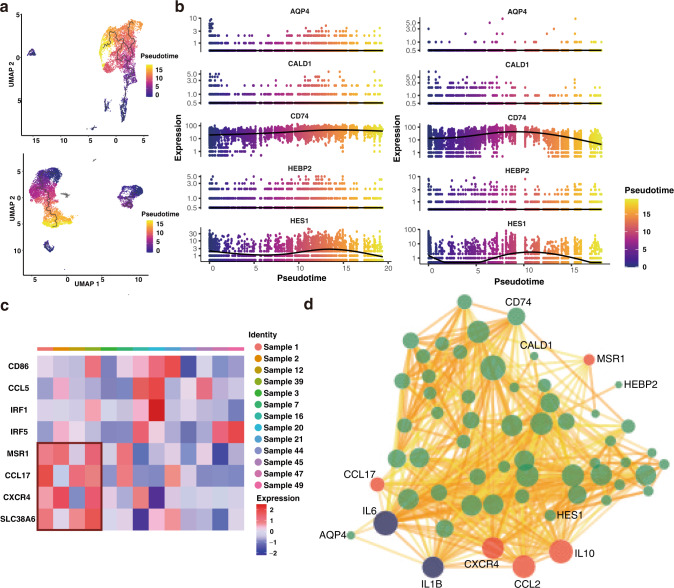
Pseudo-time trajectory analysis revealed different regulation patterns in macrophages. **a** Pseudo-time trajectory of all macrophages extracted from single-cell samples. Upper: the trajectory of the high AQP4 group. Below: the trajectory of the low AQP4 group. **b** Pseudo-time trajectory of DEGs of macrophages in high AQP4 (left) and low AQP4 group (right) extracted from single-cell samples. **c** Average expression level of markers representing M1 (CD86, IL6, IRF1, IRF5) and M2 macrophages (MSR1, CCL17, CXCR4, SLC38A6) in all single-cell samples. **d** Protein interaction network showing DEGs (green and labeled dots), M2 (red dots), and M1 markers (blue dots). The size of dots indicates the degree of connectivity, and the shades of yellow indicate the combined score.

Thus, we extracted macrophages from initially clustered scRNA-seq data (Fig. 1c, cluster “Macrophages”), split them into two groups by the same standard of tumor cells, and constructed a single-cell pseudo-time trajectory via Monocle 3 R package. Dimension reduction and batch effect removal was performed before ordering cells based on progression states. The root state of the pseudo-time trajectory was manually set with markers referencing the work of Nestorowa et al. [45], inferring they hold the characteristic of progenitors. We noted that the pseudo-time trajectory pattern of the DEGs was almost identical, except that the CD74 expression level in the low AQP4 macrophage group was slightly lower than in the high AQP4 macrophage group (Fig. 5a, b). Next, we analyzed the average expression of M1 and M2 polarized macrophage markers to evaluate the polarization profile related to the tumor immune microenvironment. Interestingly, we noticed that macrophages of the high AQP4 group tended to polarize in the direction of M2 macrophages, while those of the low AQP4 group showed no apparent tendency (Fig. 5c). This result was corroborated by flow cytometry analysis (Supplementary Fig. S3). We retrieved the functional protein association network to explore further the potential proteins interacting with AQP4 (Fig. 5d, Supplementary Table S5). Four DEGs were presented in the network, consistent with the result we derived with Seurat, demonstrating that AQP4 is associated with DEGs. In addition, we observed that many genes that upregulated in M2 polarized macrophages were also revealed in this network.

### AQP4 expression level is associated with the distribution of glioma cellular states

**Fig. 6:**
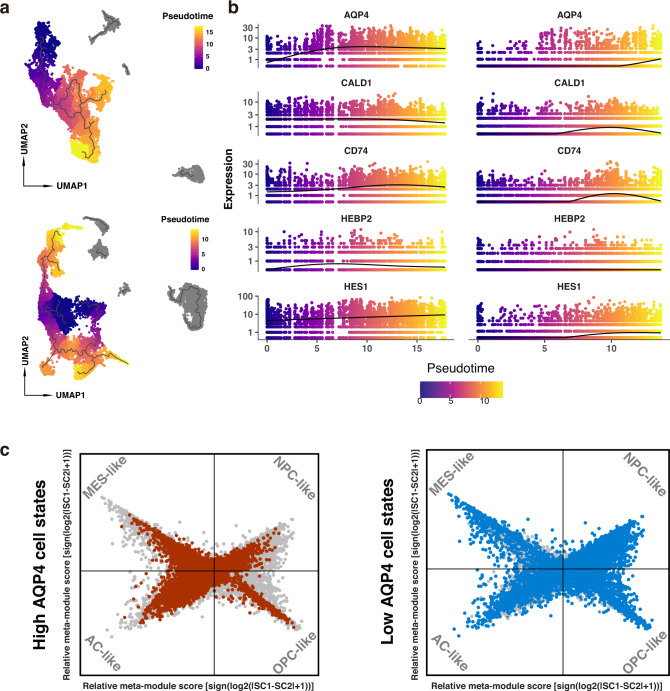
Pseudo-time trajectory analysis revealed different regulation patterns in tumor cells. **a** Pseudo-time trajectory of all glioma. Left: the trajectory of the high AQP4 group. Right: the trajectory of the low AQP4 group. Purple: early stages. Yellow: late stages. **b** Pseudo-time trajectory of DEGs of glioma cells in high AQP4 (left) and low AQP4 group (right). Different from the high AQP4 group, DEGs of the low AQP4 group did not upregulate at an early stage. **c** Two-dimensional plot visualization of different cell state scores. Red dots highlight high AQP4 populations. Blue dots represent low AQP4 populations.

Next, we focused on the association between AQP4 and the progression of gliomas. Tumor cells of high AQP4 and low AQP4 groups were separately pooled, dimension reduced, and normalized via Monocle 3 R package. The pseudo-time trajectory was performed based on the same protocol we used in Fig. 5a. We chose OLIG2 as a root-state marker, as it had long been treated as stem cells of malignant glioma [46–48], and ordered cells to visualize the development of glioma cells (Fig. 6a). Similarly, we constructed a pseudo-time trajectory pattern of the DEGs in tumor cells (Fig. 6b). Notably, DEGs are hardly expressed at the start point of the pseudo-time trajectory, i.e., cells with proliferation potential, implying that DEGs in the low AQP4 group tend to upregulate in cells with invasion potential. We asked whether this difference is related to the diversity of the microenvironment. A previous study reported that GBM malignant cells contain four different cellular states, which are plastic depending on the variety of microenvironment [49]. Based on this, the tumor cells of GBM samples were pooled to examine different cellular state proportions. Following the integrative model of GBM reported by Neftel et al. [49], we used their published algorithm to highlight oligodendrocyte-progenitor-like (OPC-like), neural-progenitor-like (NPC-like), astrocyte-like (AC-like), and mesenchymal-like (MES-like) cells (Fig. 6c). Different cellular states landscape was revealed in two groups: Most cells in GBMs of the high AQP4 group tended to be MES-like and AC-like cells while those of the low AQP4 group did not, suggesting AQP4 expression level may be associated with GBM cellular states proportions.

## Discussion

AQP4 is a glial water channel protein that regulates water homeostasis in the brain. It has been identified that alterations of the AQP4 aggregation state can influence plasma membrane dynamics, offering the potential for metastasis of glioma and changes of the tumor microenvironment. However, few previous studies have put AQP4 and the glioma immune microenvironment together, and neither have they utilized single-cell sequencing analysis to reveal the AQP4 expression profile in malignant gliomas. We performed AQP4-related analyses of different types of cells in malignant glioma samples using single-cell transcriptome analysis. Our findings discovered for the first time that AQP4 can alter the polarization tendency of tumor-associated macrophages (TAMs), associated with the immune microenvironment of glioma, consequently causing alterations in glioma cellular states.

AQP4 is generally highly expressed in brain tissues, which is determined by the properties of AQP4 and has been widely confirmed by studies [18, 19]. We further validated that its expression level is even higher in gliomas compared with normal brain tissues. We found that increased expression of AQP4 can lead to reduced OS in malignant gliomas, resulting in a poor prognosis. Previous studies have shown that high levels of AQP4 expression are not associated with OS in GBMs [22]. Consistently, our study found that high levels of AQP4 expression are not associated with OS in GBM, either. However, AQP4 expression levels were strongly correlated with OS of LGGs, i.e., LGGs with high levels of AQP4 expression may have shorter OS and are more likely to be led to poor prognosis. We speculate that this is because GBM itself has an extremely short overall survival, and the influence of AQP4 is on the mild side.

Since AQP4 is crucial in the glymphatic system and able to maintain water homeostasis in brain tissue [18, 19], we suspect that there may also be a correlation between AQP4 and edema and drug delivery. In the treatment of glioma, radiotherapy may lead to edema, and the effect of chemotherapy is dependent on drug delivery. Out of this, we further investigated the relationship between AQP4 and the difference in OS of individuals under radiotherapy and chemotherapy. We found that the level of AQP4 expression could differentially affect the OS after chemotherapy, but not that of radiotherapy. We theorized that this might be related to the regulation of permeability of the blood-brain barrier by AQP4. Previous studies have shown that enhanced phosphorylation of AQP4 can reduce water permeability and decrease the invasiveness of tumor cells. Still, its association with chemotherapeutic drug delivery has not been analyzed, and the relationship with prognosis has not been elucidated. Therefore, more validation studies are needed to address this issue.

By performing differential expression analysis on our malignant glioma samples, we identified four DEGs, CD74, HES1, CALD1, and HEBP2, significantly upregulated in the AQP4 high expression group. All four DEGs were more or less associated with the tumor microenvironment and the immune system [38–42, 50]. Since all four DEGs with similar expression patterns to AQP4 were associated with the tumor microenvironment and the immune system, we suspected that AQP4 itself might be related to the immune microenvironment of glioma. Accordingly, we performed further analysis for TAMs in our samples. We found that the TAMs in the high AQP4 group tended to become M2 macrophages, whereas the TAMs in the low AQP4 group did not demonstrate a significant polarization tendency. In the protein interaction network, many M2 macrophage markers were noticed, along with a few markers of M1 macrophages linked to AQP4, too. However, some markers of M1 macrophages in the network were linked with those of M2 macrophages in this network. IL-6, for example, is upregulated in M1 macrophages but was also reported to be able to influence the balance between M1 and M2 macrophages and contribute to inducing M2 macrophage polarization [51, 52], explaining the noticeable M2 type bias of macrophages in the high AQP4 group. A previous study has demonstrated that AQP4 blockade alleviated irradiated lung damage and inhibited activation of M2 macrophages [53]. However, similar studies related to brain tumors are absent at this moment. With trajectory analysis, we noticed that in the low AQP4 group, the expression of CD74 is upregulated earlier than AQP4, indicating that CD74 may play an upstream regulating role for AQP4. Plus, CD74 is a receptor of macrophage migration inhibitory factor (MIF). Several studies have shown that MIF/CD74 impedes microglial M1 polarization [54–56]. We suspect that AQP4 has the ability to indicate the polarization propensity of macrophages and thus the glioma immune microenvironment.

As cellular states of GBMs are plastic depending on the diversity of the microenvironment [49], we further examined the cellular states of GBMs in our single-cell samples. Our results show that samples with higher AQP4 expression levels tended to be MES-like and AC-like states. It has been proven that MES-like and AC-like cells express major histocompatibility complex significantly higher, indicating the interaction between immune cells and MES-like cells (as well as AC-like cells) [57]. Furthermore, the previous study also revealed that macrophages induce a transition of tumor cells into MES-like states, and MES-like cells are also related to a mesenchymal program in macrophages [57]. Thus, we assume the alteration of AQP4 expression levels may involve the interaction between the immune microenvironment and tumor cell states, impacting glioma’s malignancy by transitioning into MES-like states. However, our study does not yet have enough data to decipher the effects of AQP4 on the growth and development of glioma cells or TAMs, and more extensive work is warranted for further investigation.

In summary, our study provides a novel perspective to investigate the impacts of AQP4. We identified the crucial role that AQP4 plays in the immune microenvironment of malignant gliomas, suggesting that AQP4 has the potential to be a breakpoint in malignant glioma for future discovery of the immune microenvironment of glioma.

## Material and methods

### Glioma samples collection and processing

The fresh glioma samples were obtained during the surgeries in size of 1 cm × 1 cm × 1 cm. All samples were resected from tumor core area. Detailed sample information is listed in Supplementary Table S1. The usage of samples was approved by the Institutional Review Board at Nanjing Brain Hospital Affiliated to Nanjing Medical University. Informed consent was signed by each patient. Samples were stored in GEXSCOPE™ Tissue Preservation Solution (Singleron Biotechnologies, Nanjing, China) at 4 °C and transported to the laboratory within 6 h. The specimens were rinsed with Hanks Balanced Salt Solution (HBSS) three times and split into 1–2 mm pieces. The pieces were then digested with 2 ml of GEXSCOPE™ Tissue Dissociation Solution (Singleron Biotechnologies) at 37 °C for 15 min with sustained agitation. Then, the samples were filtered through 40 µm sterile strainers and centrifuged at 1000 rpm for 5 min, 4 °C. Afterward, the supernatants were discarded. The cell pellets were suspended in 1 ml phosphate-buffered saline (PBS; HyClone). 2 ml GEXSCOPE™ Red Blood Cell Lysis Buffer (Singleron Biotechnologies) was added to cell suspension at 25 °C for 10 min to remove the red blood cells. Subsequently, the solution was centrifuged at 1000 rpm for 5 min and suspended in PBS. Samples were counted with a TC20 automated cell counter (Bio-Rad).

### Single-cell RNA library construction and sequencing

Single-cell suspension was of 1 × 10^5^ cells/ml in PBS and loaded onto a microfluidic chip according to the Singleron GEXSCOPE™ Single Cell RNA-seq Library Kit (Singleron Biotechnologies). Single-cell RNA libraries were constructed following the GEXSCOPE™ protocol [58]. After construction, the libraries were initially quantified using a Qubit 2.0 Fluorometer and diluted to 1.5 ng/µl. All libraries were more than 2 nM, examined by qRT-PCR, and pooled for sequencing. The pools were sequenced on the Illumina HiSeq X10 platform with 150 bp paired-end reads.

### Single-cell RNA library pre-processing

Raw reads were processed to generate gene expression profiles using the standard internal pipeline based on the Cell Ranger toolkit (version 2.1.1). The raw base call (BCL) files were used to generate the FASTQ files with the “mkfastq” command. After read 1 without poly T tails were removed, cell barcode and unique molecular identifiers (UMIs) were extracted. Adapters and poly-A tails were trimmed (fastp V1) before aligning the read 2 to GRCh38 Ensemble build 92 genomes (fastp 2.5.3a and featureCounts 1.6.2). Reads with the same cell barcode, UMIs, and genes were grouped to calculate the number of UMIs per gene per cell. The UMI count tables of each cellular barcode were used for further analysis.

### Single-cell RNA-seq data quality control and preparation

All the scRNA-seq data were loaded and merged into one object with Seurat [59, 60]. The following standards ruled out Low-quality cells: low feature counts (<200), high feature counts (>5000), and high mitochondrial content (>30%). The built-in SCTransform algorithm processed the Seurat object to perform sample integration, gene normalization, and data scaling. The “RunPCA” function was used to reduce dimension.

### Cell clusters annotation

Unbiased cell type recognition was performed by SingleR [61] with two comprehensive human reference data sets, one from the Human Primary Cell Atlas [62] and one from the Blueprint [63] and ENCODE [64] projects. Cell type recognition was also verified by known cluster-specific differentially expressed genes.

### Copy number variations inference

Copy number variations were inferred by inferCNV [28]. 10X counts matrix was extracted from the Seurat object and input to the “CreateInfercnvObject” function, along with the annotation file extracted from the Seurat object metadata and the genome position reference data set obtained from the GENCODE project (version 36). Then, the inferCNV object was processed with the “infercnv::run” function with dynamic threshold denoising to infer copy number variations.

### Pseudo-time trajectory inference

We use monocle3 to perform pseudo-time trajectory inference. Briefly, we transformed the Seurat object into the monocle3 required “cell_data_set”. The batch effect was removed with cell alignment by the “align_cds” function. Dimension was reduced by the “reduce_dimension” function using UMAP. The pseudo-time trajectory was inferred by the “learn_graph” and “order_cells” functions using DDRThree. We chose cells with the most OLIG2 expression level as the “root_state”.

### Statistical analysis

Statistical analysis was performed on the IBM SPSS® software platform. The statistical significance threshold was set to *p* < 0.05. All significance was performed by *t*-test.

### General gene expression profile and survival analysis

We use GEPIA2 platform [65] to analyze the RNA sequencing expression data of glioma and normal samples from the TCGA and the GTEx projects, respectively. Gene expression profile across samples was calculated by the “General Information” pipeline. Overall survival Kaplan–Meier curve plot was generated by the “Survival Analysis” pipeline.

### Functional protein association network analysis

The protein interaction network was built based on STRING database v11 [66]. The TSV format network files were imported to Cytoscape v3.8.2 [67] and processed by network analyses. The network was visualized with an organic layout. The size of nodes was adjusted according to the degree of connectivity.

### Immunofluorescence

The samples were collected from formalin-fixed paraffin-embedded sections of primary GBMs. First, the sample slides were incubated with the first primary antibody against AQP4 (#16473-1-AP; Proteintech; 1:200) overnight at 4 °C. Then, the samples were incubated with the first corresponding secondary antibody for 50 min at room temperature away from light. Next, the samples were incubated with the second primary antibody CD74 (#GB12179; Servicebio; 1:200) overnight at 4 °C and with the second corresponding secondary antibody for 50 min at room temperature away from light. DAPI is used for counterstaining to display nuclei. The same protocol was applied to AQP4(#16473-1-AP; Proteintech; 1:2000) and the rest of DEGs: HES1 (#GB112254; Servicebio; 1:5000), HEBP2 (#A71824-050; Epigentek; 1:1000), CALD1 (#HPA017330-S; Atlas antibodies; 1:2000). At last, the sample slides were imaged using Nikon Imaging System. Multispectral images were processed and analyzed using 3DHISTECH CaseViewer software.

## Supplementary information

Supplementary Figure S1

Supplementary Figure S2

Supplementary Figure S3

Supplementary Table S1

Supplementary Table S2

Supplementary Table S3

Supplementary Table S4

Supplementary Table S5

## Data Availability

The scRNA-seq datasets presented in this study can be found in GEO repositories at: https://www.ncbi.nlm.nih.gov/geo/, GSE135045 and GSE167960.
