# Correction to: Single-cell RNA sequencing reveals changes in glioma-associated macrophage polarization and cellular states of malignant gliomas with high AQP4 expression

**DOI:** 10.1038/s41417-023-00592-4

**Published:** 2023-02-09

**Authors:** Ran Wang, Lu Peng, Yong Xiao, Qi Zhou, Zhen Wang, Lei Tang, Hong Xiao, Kun Yang, Hongyi Liu, Li Li

**Affiliations:** 1 Department of Neurosurgery, The Affiliated Brain Hospital of Nanjing Medical University, Nanjing, Jiangsu China; 2 Department of Clinical Laboratory, The Affiliated Brain Hospital of Nanjing Medical University, Nanjing, Jiangsu China; 3 Department of Neuro-Psychiatric Institute, The Affiliated Brain Hospital of Nanjing Medical University, Nanjing, Jiangsu China; 4 Department of Laboratory Medicine, Shanghai General Hospital of Nanjing Medical University, Shanghai, China

**Keywords:** CNS cancer, Tumour heterogeneity

Correction to: *Cancer Gene Therapy* 10.1038/s41417-022-00582-y, published online 04 January 2023

In the article “Single-cell RNA sequencing reveals changes in glioma-associated macrophage polarization and cellular states of malignant gliomas with high AQP4 expression” by Wang et al., Cancer Gene Ther., 2023, 10.1038/s41417-022-00582-y, a correction was needed.

The order of the author list should be “Ran Wang, Lu Peng, Yong Xiao, Qi Zhou, Zhen Wang, Lei Tang, Hong Xiao, Kun Yang, Hongyi Liu, Li Li”.

The order of the corresponding authors’ email addresses should be “email: doctoryk@njmu.edu.cn; njnkyylhy@163.com; annylish@126.com”.

The claim of the corresponding authors should be “Correspondence to Kun Yang, Hongyi Liu or Li Li.” in the HTML version, and “Correspondence and requests for materials should be addressed to Kun Yang, Hongyi Liu or Li Li.” in the PDF version.

The original article has been corrected.

