# Supplementary material for: Single-cell RNA sequencing reveals changes in glioma-associated macrophage polarization and cellular states of malignant gliomas with high AQP4 expression
Source: Cancer Gene Ther. 2023 Jan 4;30(5):716–26. doi: 10.1038/s41417-022-00582-y (PMC10191842; doi:10.1038/s41417-022-00582-y)
Supplement: Supplementary file 1 — Supplementary Figure S1 [file 41417_2022_582_MOESM1_ESM.docx]

**a**

**
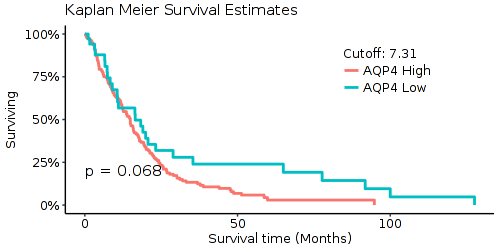
**

**b**

**
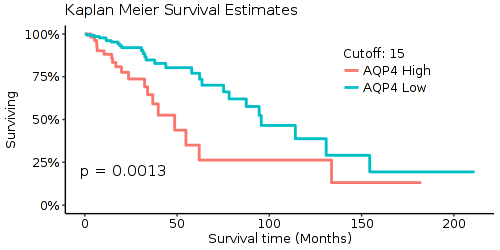
**

**c**

**
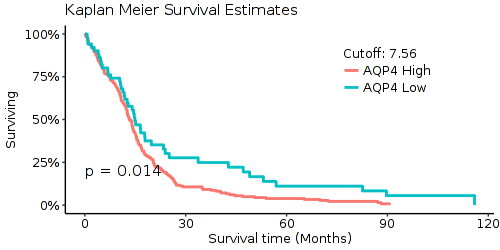
**

**d**

**
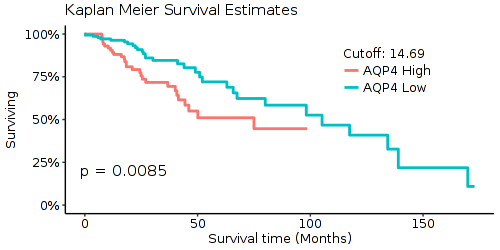
**

**e**

**
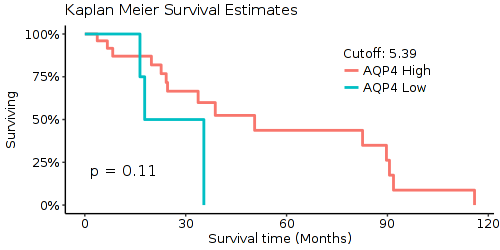
**

**f**

**
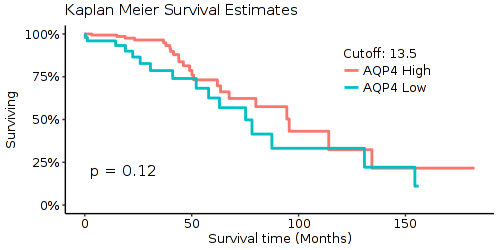
**

**g**

**
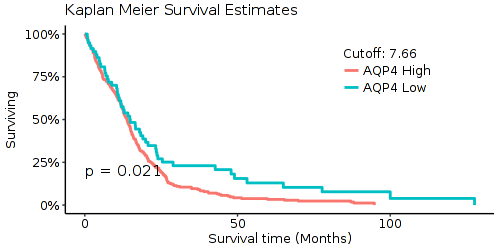
**

**h**


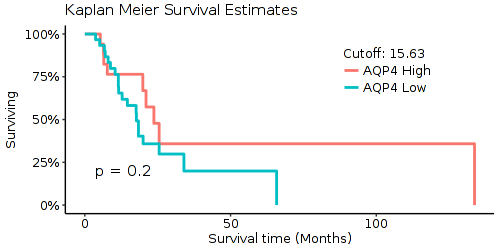


**Supplementary Figure S1**

**a** Survival curve of female patients with glioblastoma.

**b** Survival curve of female patients with lower grade glioma.

**c** Survival curve of male patients with glioblastoma.

**d** Survival curve of male patients with lower grade glioma.

**e** Survival curve of glioblastoma patients with IDH mutant type.

**f** Survival curve of lower grade glioma patients with IDH mutant type.

**g** Survival curve of glioblastoma patients with IDH wild type.

**h** Survival curve of lower grade glioma patients with IDH wild type.
