# Supplementary material for: Single-cell RNA sequencing reveals changes in glioma-associated macrophage polarization and cellular states of malignant gliomas with high AQP4 expression
Source: Cancer Gene Ther. 2023 Jan 4;30(5):716–26. doi: 10.1038/s41417-022-00582-y (PMC10191842; doi:10.1038/s41417-022-00582-y)
Supplement: Supplementary file 2 — Supplementary Figure S2 [file 41417_2022_582_MOESM2_ESM.docx]

|   **a** |
| --- |
|   **b** |
| **Supplementary Figure S2**  **a** Heatmap showing hallmark gene set enrichment score of each sample.  **b** Gene ontology enrichment of differentially expressed genes between high and low AQP4 groups. |
