# Supplementary material for: Single-cell RNA sequencing reveals changes in glioma-associated macrophage polarization and cellular states of malignant gliomas with high AQP4 expression
Source: Cancer Gene Ther. 2023 Jan 4;30(5):716–26. doi: 10.1038/s41417-022-00582-y (PMC10191842; doi:10.1038/s41417-022-00582-y)
Supplement: Supplementary file 3 — Supplementary Figure S3 [file 41417_2022_582_MOESM3_ESM.docx]

| 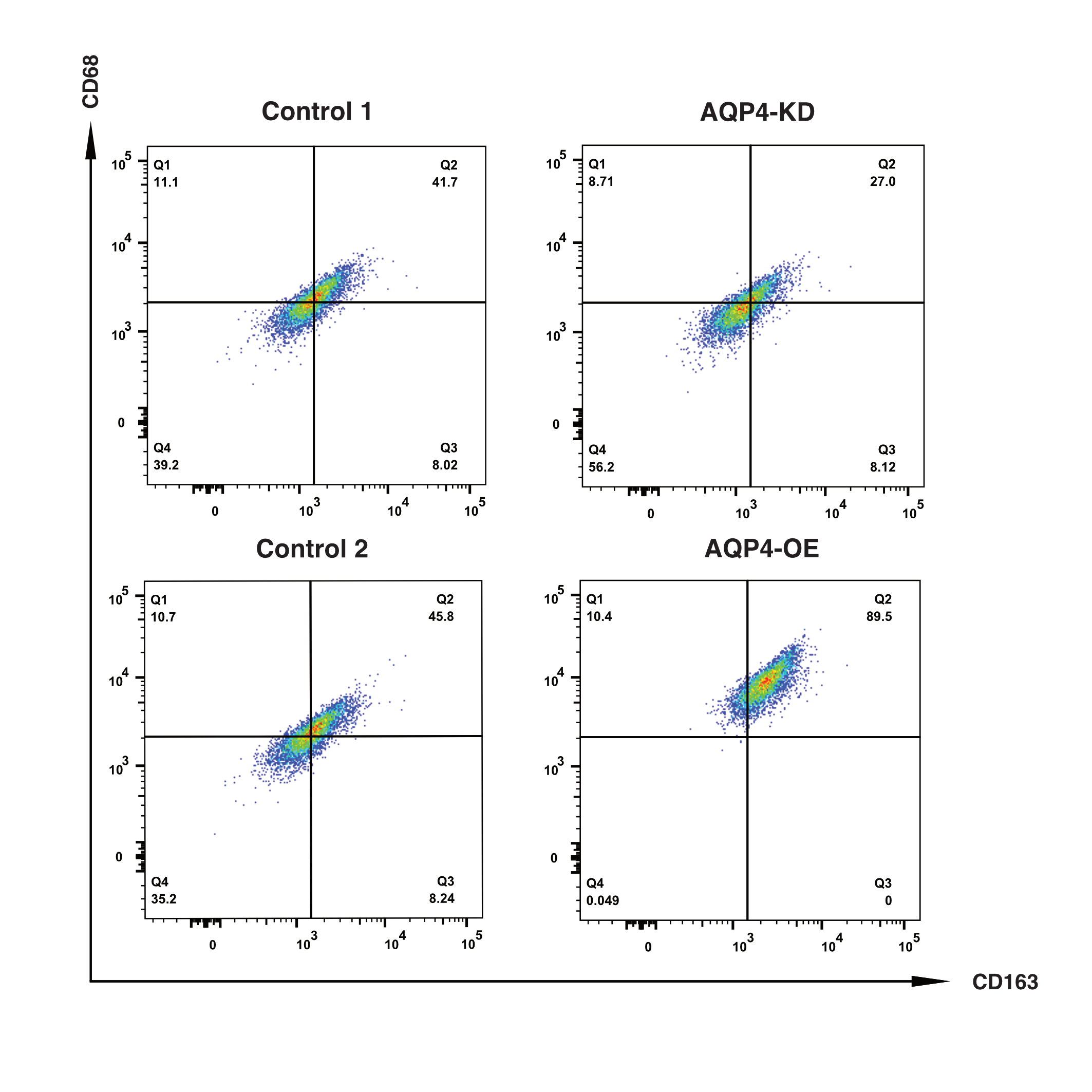 |
| --- |
| **Supplementary Figure S3**  Flow cytometric analysis of macrophages co-cultured with cell-free supernatants of U-251, U-251 with AQP4 knockdown, and U-251 with AQP4 over-expressed cells. |
